# Research Progress on miRNAs and Artificial miRNAs in Insect and Disease Resistance and Breeding in Plants

**DOI:** 10.3390/genes15091200

**Published:** 2024-09-12

**Authors:** Zengfeng Ma, Jianyu Wang, Changyan Li

**Affiliations:** 1 Rice Research Institute, Guangxi Academy of Agricultural Sciences, Guangxi Key Laboratory of Rice Genetics and Breeding, State Key Laboratory for Conservation and Utilization of Subtropical Agro-bioresources, Nanning 530007, China; mazengfeng@gxaas.net; 2 Food Crops Institute, Hubei Academy of Agricultural Sciences, Wuhan 430070, China; 202221107012051@stu.hubu.edu.cn; 3 School of Life Sciences, Hubei University, Wuhan 430062, China

**Keywords:** microRNA, plant, insect resistance, disease resistance, breeding

## Abstract

MicroRNAs (miRNAs) are small, non-coding RNAs that are expressed in a tissue- and temporal-specific manner during development. They have been found to be highly conserved during the evolution of different species. miRNAs regulate the expression of several genes in various organisms, with some regulating the expression of multiple genes with similar or completely unrelated functions. Frequent disease and insect pest infestations severely limit agricultural development. Thus, cultivating resistant crops via miRNA-directed gene regulation in plants, insects, and pathogens is an important aspect of modern breeding practices. To strengthen the application of miRNAs in sustainable agriculture, plant endogenous or exogenous miRNAs have been used for plant breeding. Consequently, the development of biological pesticides based on miRNAs has become an important avenue for future pest control methods. However, selecting the appropriate miRNA according to the desired target traits in the target organism is key to successfully using this technology for pest control. This review summarizes the progress in research on miRNAs in plants and other species involved in regulating plant disease and pest resistance pathways. We also discuss the molecular mechanisms of relevant target genes to provide new ideas for future research on pest and disease resistance and breeding in plants.

## 1. Introduction

**Figure 1:**
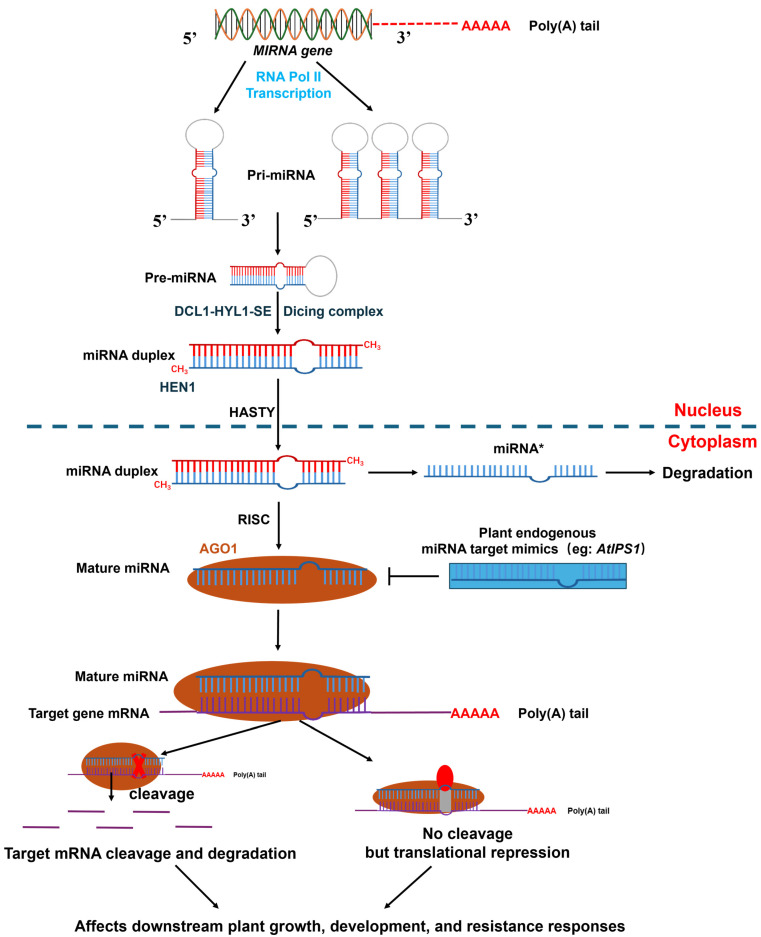
Molecular mechanisms related to the regulation of endogenous miRNA expression in plants. Primary microRNA (pri-miRNA) is transcribed by Pol II from miRNA-encoding genes. In the nucleus, the RNase III family enzyme DCL1, along with HYL1 and SE, processes pre-miRNA into miRNA/miRNA double strands. The miRNA/miRNA* double strand is methylated at its 3′ end by the miRNA methyltransferase HEN1. Once methylated, the miRNA/miRNA* double strand is transported from the nucleus to the cytoplasm through HASTY. In the cytoplasm, the miRNA/miRNA* double-stranded guide strands are incorporated into RISC. This process is involved in miRNA degradation, as well as miRNA-mediated gene silencing through target cleavage and translational repression [6].

Mature microRNAs (miRNAs) are non-coding RNA molecules that are approximately 20–24 nt in length. These can regulate gene expression at the post-transcriptional level by inhibiting mRNA translation or promoting mRNA degradation [1]. miRNA sequences have remained highly conserved during gene evolution across different species, with miRNA expression patterns exhibiting tissue and temporal specificity during biological development [2,3]. Notable differences exist between plant and animal miRNAs. For instance, plant miRNAs can bind to various positions of an mRNA sequence, whereas animal miRNAs typically bind to the 3′-untranslated region of the target mRNA. Unlike plant miRNAs, only a few sequences near the 5′-end of animal miRNAs are completely complementary to target genes, and the complementary degree of other sequences is comparatively low. Therefore, miRNAs in animals primarily function by inhibiting translation [4,5]. Based on these observations, it can be speculated that the functional role of miRNAs in plants can produce more interesting possibilities (Figure 1).

During their growth and development, plants experience various stresses, including insect pest infestation and diseases. Thus, exploring the relationship between miRNAs and plant stress responses can aid future studies and inform the development of cultivation strategies to produce new plant varieties with stronger stress resistance. Currently, bioinformatics analysis and biological experiments are the primary methods adopted to study the involvement of miRNAs in plant biological stress. The related studies have identified various miRNAs as contributors to disease and pest resistance in different model plants, including *Arabidopsis thaliana*, *Nicotiana tabacum*, and *Brachypodium distachyon*, as well as crops such as *Oryza sativa*, *Zea mays*, and *Hordeum vulgare*. Information regarding such miRNAs can be found in the miRBase Sequence Database (http://www.mirbase.org/ (accessed on 1 July 2024)), the largest miRNA-related database that contains >48,860 mature miRNA sequences from 271 species, including those of animals, plants, and microorganisms [7].

With continuous research and technological advances, the mechanisms by which miRNAs and their target genes regulate plant stress responses have become increasingly clear. miRNAs, which comprise a large family, are characterized by numerous target genes and complex regulatory mechanisms. In both plants and animals, miRNAs act as central components of the regulatory network that controls responses to external stresses. They can regulate a large number of target genes, which are often involved in complex biological processes and signaling pathways. Therefore, even a small change in a miRNA can trigger a butterfly effect. Functional verification of miRNAs in response to stress and their role in plant molecular regulatory networks will provide a strong scientific basis for further research on plant resistance to diseases and insect pests.

## 2. Progress in miRNA Research on Insect Resistance and Breeding in Plants

Insects can cause considerable crop losses directly or indirectly. Chemical control methods such as pesticide use can increase crop yields, but most pesticides are harmful to consumer health and the environment. Thus, there is a need for safer methods, such as the manipulation of plant and insect endogenous miRNAs.

### 2.1. Plant Endogenous miRNAs

The application potential of endogenous miRNA in developing resistance to insect pests in plants has not been investigated in depth. However, some studies have proven the use of plant endogenous miRNAs is a viable and safe method (Table 1).

For example, it has been demonstrated that AtmiR156 targets the squamosa promoter binding protein-like 9 (*SPL9*) transcription factors in *A. thaliana* and that SPL9 interacts with jasmonate-zim-domain protein 3 (*JAZ3*) in the jasmonic acid (JA) signaling pathway. This leads to the accumulation of JAZ3 and a gradual increase in SPL9 levels, weakening the JA response and enhancing insect resistance in plants [8]. Furthermore, OsmiR156 negatively regulates *N. lugens* (brown planthopper [BPH]) resistance by increasing JA expression in rice and silencing OsmiR156 improves BPH resistance [11]. OsmiR159 reportedly influences BPH resistance by regulating its target; repression of *OsGAMYBL2* by OsmiR159 inhibits *GS3* gene expression, thus increasing BPH resistance [12]. Overexpression of an OsmiR396 target gene, growth-regulating factor 8-like (*OsGRF8*), increases the flavonoid content in plants and confers BPH resistance. On the other hand, *OsGRF8* directly regulates the expression of BPH-responsive flavanone 3-hydroxylase (*OsF3H*) in the flavonoid biosynthesis pathway, revealing a new BPH resistance mechanism mediated by the OsmiR396–*OsGRF8-OsF3H*–flavonoid pathway [13].

A previous study demonstrated that overexpression of ghr-miR166b in *G. hirsutum* targets the *Bemisia Tabaci* ATP synthase gene, conferring resistance against infection [9]. Meanwhile, another study found that the differential expression of miR159a, miR160a, and miR393a in *Chrysanthemum morifolium* is related to its response to aphids. Moreover, miR164, miR167, miR390, and miR393 are known to be expressed in aphid-resistant *A. thaliana* and *Cucumis melo* upon aphid invasion [17].

Recently, it was discovered that the expression of rice OsmiR-162a is induced to initiate a defense response upon BPH invasion. Consequently, BPH reproduction and development are negatively regulated through cross-border silencing, and BPH host selectivity is reduced by inhibiting the α-linolenic acid metabolic pathway of rice [13]. Utilizing plant endogenous miRNAs for the development of insect resistance in plants is undoubtedly a more environmentally friendly approach compared to chemical control methods.

### 2.2. Insect Endogenous miRNAs

Besides miRNAs related to endogenous insect resistance in plants, numerous miRNAs directly involved in insect development have been used to advance insect resistance research. miRNAs affect hormone metabolism, molting behavior, tissue degradation, and reconstruction during insect metamorphosis [18,19,20]. The expression levels of multiple miRNAs are closely related to molting and metamorphosis during *Drosophila* development. For example, key genes in the ecdysone functional pathway can significantly upregulate miR-100, miR-125, and let-7 expression and inhibit miR-34 expression to jointly maintain the molting process [21,22]. mir-14 can target the ecdysone receptor gene in *Drosophila*, forming a feedback regulation loop to maintain the appropriate level of ecdysone and efficiently initiate or terminate the corresponding molting process [23]. In *Bombyx mori*, miR-14 can affect multiple gene expressions and steps of the ecdysone regulatory network to maintain growth and development [24,25]. miR-9a controls apoptosis during *Drosophila* wing development via the negative regulatory transcription factor LIM-only protein (dLMO) to regulate wing shape [26]. During the differentiation and development of wings in BPHs, miR-9a can respond to host nutrition levels and the signal regulation of the insulin receptor gene to promote the development of populations with either long or short wings by regulating ultrabithorax expression [27].

Insect endogenous miRNAs have previously been utilized to confer insect resistance in plants. For example, a miRNA and a hairpin RNA (hpRNA) for acetylcholinesterase 2 (*MpAChE2*) have been designed and transferred into tobacco plants to obtain T_2_-generation transgenic plants consumed by aphid *Myzus persicae*. *M. persicae* that ingested the transgenic miRNA-containing tobacco plants displayed lower fecundity than those that ingested the transgenic hpRNA-containing plants, indicating that the miRNA displayed a stronger anti-insect effect than hpRNA [28]. In *H. armigera* larvae that were fed transgenic tobacco plants expressing a miRNA targeting the chitinase gene based on the endogenous miR-24, the expression of the chitinase gene in the larvae was substantially decreased, leading to molting cessation and larval death [10].

Taken together, these findings indicate that the regulatory effect of miRNAs on the physiological and biochemical processes of insects is conserved and warrants further verification for the development of pesticides in future studies. Drug development based on miRNA expression regulation should consider the advantages of both specificity and broad-spectrum action.

## 3. Progress in miRNA Research on Disease Resistance and Breeding in Plants

The miRNAs involved in plant disease resistance have been extensively examined (Table 2). miR393, which restricts *Agrobacterium tumefaciens* (*C58*) growth by degrading auxin receptors and inhibiting auxin signals, was the first miRNA found to be involved in host–pathogen interactions [29]. Subsequently, several miRNAs related to plant biological stress resistance have been identified, and their functions have also been analyzed. miR393 and miR393* are a pair of miRNAs that regulate two pathways through different target proteins; the former targets auxin signaling, whereas the latter targets membrin 12 (*MEMB12*), which encodes soluble N-ethylmaleimide-sensitive factor activating protein receptor (SNARE). In turn, the effector-triggered immune response induced by effectors is promoted via the regulation of pathogenesis-related protein 1 (*PR1*) expression [30]. miR393 is also closely related to plant hormone signaling pathways and responds to bacterial infection by negatively regulating the expression of auxin receptor genes (transport inhibitor response 1 [TIR1], auxin signaling F-box 2 [*AFB2*], and auxin signaling F-box 4 [*AFB4*]) [31,32,33]. Rice blast fungus is caused by fungi (e.g., *Magnaporthe grisea*) and affects rice production; miR398b can regulate H_2_O_2_ accumulation through several superoxide dismutase (*SOD*) genes, thereby influencing resistance to rice blast [34]. H_2_O_2_ accumulation can cause reactive oxygen species (ROS) explosion and affect the expression of several genes, regulating growth and inducing programmed cell death (PCD) [35]. It has been reported that the expression of adult plant resistance (APR) to stripe rust in the wheat variety XZ9104 (XZ) is related to extensive allergic cell death and local production of ROS in host cells, and two novel miRNAs (1136-P3 and PN-2013) were found to be associated with this effect. PN-2013 promotes H_2_O_2_ accumulation by regulating the expression of the target gene monodehydroascorbate reductase (*MDHAR*), which decreases the activities of ascorbate peroxidase and catalase and increases the expression of several disease-resistance genes [36]. miR528 activates the antiviral pathway in rice by regulating *L*-ascorbate oxidase and inhibiting ROS accumulation mediated by it, thereby reducing cell death and host plant damage [37]. Overall, understanding the regulatory mechanisms of miRNAs and their target genes is important for improving disease resistance and yield in plants.

In addition to regulating plant growth and development by acting on transcription factors, miRNAs participate in the “struggle” between plants and pathogens. Pathogenic microorganisms inhibit host immune defenses and exacerbate pathogenic processes by expressing or inducing host plants to express specific miRNAs [47]. For example, *P. syringae* inhibits the transcription of self-defense genes, such as atypical receptor-like pseudokinase 1/2 (*ARLPK1/2*) and SERRATE when infecting *A. thaliana*, by inducing miR-863 overexpression in the host, thereby shutting down the plant immune process [48]. Cucumber mosaic virus (CMV) encodes protein 2b, which competitively binds to the host argonaute 1 (AGO1) protein, preventing plants from processing/modifying their own miRNAs and silencing host defense responses mediated by miRNAs [49]. In contrast, plants resist pathogenic microorganisms by upregulating or downregulating endogenous miRNA expression to activate their own immune system and pathogen resistance pathways. For example, miR482 can silence members of the NBS-LRR gene family. NBS-LRR proteins are a type of resistance (R) proteins of the plant innate immune system involved in effector-triggered immunity. When tobacco is infected by the tobacco mosaic virus, it reduces the expression level of its own miR-482 to promote that of NBS-LRR genes, thereby resisting viral infection. [42]. The 2b protein of CMV makes ZmmiR168 cut *AGO1* through complex interactions with AGO1 and dicer-like 1 (DCL1), increases *AGO1* expression, and indirectly regulates the functions of other miRNAs [49].

Using gene editing or transgenic technology, plants can be genetically modified to overexpress specific miRNAs, thereby suppressing key pathogenicity genes. This technique has been implemented in the development of rice, tomato, tobacco, and other crops [50,51].

Plant pathogens are destructive biological factors that adversely affect plant growth and development; however, plants possess complex mechanisms to cope with this process [52]. Recently, the involvement of miRNAs has been demonstrated in plant diseases caused by viral, bacterial, and fungal pathogens. In *A. thaliana*, lowering miR171 levels can increase the expression of most target genes, resulting in increased susceptibility to viral infection [31,32]. Furthermore, AtmiR160a, AtmiR398b, and AtmiR773 are involved in plant responses to bacterial diseases [53], while other plant miRNAs play an important role in plant responses to fungal diseases. An analysis of changes in miRNA expression in cotton roots infected with Verticillium wilt revealed that the expression of at least 65 miRNAs was found to be altered, with most being non-conservative miRNAs [39]. A study on the mechanism of resistance to sheath blight in maize found upregulated expression of miR168 after infection. The expression of the target gene *AGO1* was speculated to decrease, accumulating numerous other resistance target genes through negative feedback regulation, inducing resistance to pathogen infection [46]. Moreover, the AGO protein plays a key role in plant disease resistance and target gene expression regulation by miRNAs [54,55]. Overall, miRNAs play important roles in plant resistance to diseases.

The important regulatory role of miRNAs in resistance to plant diseases and their ability to target and regulate disease resistance/susceptibility genes provides a new perspective for studying mechanisms underlying resistance to plant diseases [46,55,56]. Recently, miRNAs were isolated from various plants, including wheat, rice, and *Arabidopsis*. In *A. thaliana*, a decrease in miR398 and miR773 expression increased the transcription of SOD and methyltransferase genes, which in turn improved resistance to plant diseases [57]. miR167 and miR396 have been shown to affect the citrus defense system [58]. In plant disease resistance, R proteins recognize specific pathogen-related effectors and activate defense responses to inhibit rapid pathogen growth [59,60]. Tomato miR482, related to the disease resistance gene *R*, can interact with 58 *R* genes (gene families related to disease resistance), especially in the CC-NBS-LRR domain [61]. In response, miRNA expression is induced or inhibited to regulate the expression of genes involved in the disease response. Tobacco miR6019 and miR6020 act on Toll/interleukin-1 receptor-like-NBS-LRR domain-type proteins [58]. Regarding fungal diseases, downregulation of miR2118 expression in cotton roots infected with *Verticillium dahlia* results in the accumulation of numerous target proteins known to improve the defense ability of plant roots. miR166 and miR159 expression is upregulated in cotton infected with *V. dahliae*, causing mycelium-specific silencing [27]. Further, it has been found that the fungal virulence genes Ca^2+^-dependent cysteine protease (*Clp-1*) and trichodermin C-15 hydroxylase (*HiC-15*) could be recognized and degraded by miRNAs [40]. Following poplar tree infection with a canker pathogen, miR1447 expression reportedly increased, whereas miR1448 and miR472 expression decreased. All these miRNAs regulate disease-resistance protein expression [62]. For example, miR7695 overexpression in rice can enhance resistance to rice blast [63]. miR160a and miR398b overexpression results in increased resistance to *M. grisea* at the infection site in transgenic rice [64]. In wheat infected with powdery mildew, the expression of miR156, miR159, and miR164 is upregulated, whereas that of miR393, miR444, and miR82 is downregulated [65]. However, further experiments are needed to verify the regulation of miRNA expression and the characteristics of target genes in different plants and fungal infections [66]. Thus, studying the interaction between miRNAs and target genes is important to improve disease resistance and disease control in plants [56,63,67].

In summary, the expression of several resistance genes is regulated by and linked to other genes by miRNAs. An in-depth study on the molecular mechanisms by which miRNAs regulate their target gene expression in crop disease resistance has laid the foundation for breeding crops with disease resistance. Furthermore, miRNAs can be easily detected and can be used as molecular markers for disease-resistance breeding; thus, they provide novel avenues of research for breeding new disease-resistant crop varieties.

## 4. Application of Artificial miRNAs (amiRNAs) in Research on Plant Resistance

amiRNAs are based on the design of an endogenous miRNA precursor, which utilizes the miRNA precursor as the basic skeleton. A miRNA precursor produces only one sRNA bivalent (specifically, a miRNA/miRNA* bivalent); replacement of the miRNA/miRNA* bivalent region sequence by a specific miRNA complementary to the target gene sequence results in an amiRNA with novel functions [68]. If the secondary reversion structure of the amiRNA precursor remains intact, its function is consistent with that of the endogenous miRNA precursor [69,70]. Thus, amiRNAs can be designed by selecting one or more target genes of interest to analyze and identify gene functions. Concurrently, amiRNA sequences should have low homology with the plant genes selected for silencing to avoid off-target effects [51,71]. In addition, plant amiRNAs have high specificity, similar to that of endogenous miRNAs [50,72]. Therefore, an optimized amiRNA sequence can specifically silence one or more target transcripts. amiRNAs specifically designed for genes in plants or insects have been extensively reported in research on disease or insect resistance (Table 3).

Notably, expressing insect pre-miRNAs in transgenic plants is more effective than expressing plant miRNA precursors in order to prevent recognition and processing by plant RNA interference (RNAi) core mechanisms. The upper and lower stem structures of pre-miRNAs in insects are more resistant to *C. suppressalis* than those of the miRNA csu-novel-260 in transgenic rice plants [78], which may explain the efficacy of insect pre-miRNAs. A recent study reported that osa-miR162a overexpression in rice can transversely silence the target of the rapamycin gene (*NlTOR*) in BPH, affecting its reproductive development [82].

miRNAs can cause insect-specific gene disorders by regulating the expression of their target genes, resulting in abnormal development, death, or reduced drug resistance in insect pests. When expressed in plants, amiRNAs target and degrade the genes of invasive insects, thereby conferring the plant with insect resistance [83]. For example, plant chitinases can destroy insect exoskeletons and play important roles in pest control. Agrawal et al. found that amiR24 targets chitinase and causes the death of cotton bollworms by interfering with the molting process. Therefore, transgenic tobacco plants overexpressing amiR24 exhibit insecticidal properties [10]. In summary, amiRNAs also present great potential in plant resistance applications.

### 4.1. Application of amiRNAs for Insecticidal Effects in Plants

In a previous study, the mortality of two transgenic tobacco plants expressing HaAce1-amiR1 and fed *H. armigera* larvae was 25% higher than that of the control plants. Moreover, the larvae-fed plants displayed a 20% higher adult deformity rate than the control plants [75]. Precursor miR-166b sequence expression in *G. hirsutum* reportedly leads to a substantial increase in the mortality of *B. tabaci*, which feeds on *G. hirsutum* leaves [9]. Injection of miRNA mimics into *C. suppressalis* larvae cause malformation and lethality; specifically, miRNA mimics target miR-14, which targets the ecdysone receptor and spook, two key genes in the insect ecdysone signaling network. For example, a previous study reported a mortality of 60% for *C. suppressalis* at 3 days after being fed transgenic rice from two families with high miR-14 expression [24]. The transgenic rice plants expressing the endogenous miRNA of *C. suppressalis* showed significant growth-inhibition effects on *C. suppressalis* larvae. The growth period of *C. suppressalis* that was fed with the csu-novel-miR-15 transgenic rice was delayed by at least 4 days, but no obvious lethal effect was observed [79]. A previous study showed that transgenic rice expressing the endogenous miRNA csu-novel-miR-53 from *C. suppressalis* significantly inhibited its growth. The transgenic rice stem borers displayed a 7-day longer average pupation time and a 43.2% lower eclosion rate than the control group [80]. Thus, the introduction of amiRNAs into plants can effectively reduce the damage of pests to plants, which demonstrates the potential application of amiRNAs as a biological pesticide similar to BT protein and dsRNA for controlling pests in vitro.

### 4.2. Application of amiRNAs for Disease Resistance in Plants

To effectively avoid the mis-effects of traditional RNAi methods, amiRNAs are selected to generate only one segment of 21 nt, complementary to the host genome. Additionally, there is no risk of recombination between amiRNA and the non-target viral genomes of infected plants, which enables the development of safer, more efficient, and broad-spectrum antiviral transgenic plants using amiRNA technology to target viral genes. Most virus infections in the field are mixed infections, wherein the viruses mutate rapidly, leading to amiRNA mismatches. Thus, amiRNAs can be designed to target the conserved regions of viruses, effectively inhibiting the growth of most viral strains and maintaining disease resistance even after virus mutation. This method prolongs the lifespan of plant varieties and prevents the accumulation of new virus variants, which is of significance for the cultivation of long-lasting and broad-spectrum virus-resistant varieties [77,84].

The amiRNA technology was first applied to mammalian cell lines [85]. The first application was in plants, *A. thaliana* [86]. Since then, specific silencing of target gene expression using amiRNAs has been reported in *Arabidopsis*, tobacco, tomato, rice, eggplant, and cassava [73,77,87,88,89,90,91,92]. amiRNAs have been widely studied and could potentially have a great influence on inducing disease resistance in plants, particularly resistance to plant viruses [50,73]. In a previous study, the conserved fragments of the *A. thaliana* miR159a skeleton were replaced with *HC-Pro* of the turnip mosaic virus and *P69* of the turnip yellow mosaic virus to construct two amiRNA vectors. The two amiRNAs specifically silenced the target gene expression, an effect that was stably passed on to their offspring [73]. In other studies, transgenic tomato plants were transformed with an amiRNA vector targeting the CMV-silencing suppressor 2b gene, which effectively induced resistance to CMV infection [74,76]. In addition, if the precursor skeletons of amiRNAs using different viral genes are genetically transformed into the same plant, resistance to multiple viruses can be achieved simultaneously. For example, when amiRNAs targeting the silencing suppressors HC-Pro from potato virus Y (PVY) and p25 from potato virus X (PVX) were introduced into *N. tabacum*, the transgenic tobacco displayed significantly improved resistance to both PVY and PVX [70]. Researchers have designed amiRNAs based on the PVY and tobacco etch virus Nib and CP genes and found that the transgenic tobacco plants were resistant to both viruses [77]. Considering the occurrence of different diseases in the field, the miRNA expression vector could be constructed according to the conserved sequence of the pathogenic genes to broaden the disease resistance spectrum of crops [91].

However, amiRNA technology has encountered obstacles in agricultural applications. One example is that viruses can mediate changes in the conserved target regions by means of a mutation, resulting in mis-effects [92,93]. A possible solution for this is the expression of multiple amiRNAs based on multiple highly conserved regions of the virus. For example, multiple amiRNA interference vectors targeting the 2b protein-coding region and 3′ non-coding conserved region of CMV have been designed to generate transgenic *A. thaliana* highly resistant to the three CMV subgroups [94].

The designability and target specificity of amiRNA technology fully reflect the applicability and versatility of this technology in plant disease resistance breeding. With the discovery of more endogenous miRNAs and the in-depth study of their mechanism of action, amiRNA technology will continue to improve and play a greater role in the field of plant disease.

## 5. Prospects and Conclusions

### 5.1. Applications of RNAi Technology in Crop Production

The frequent occurrence of diseases and insect pests has been a persistent issue that hinders agricultural development. Chemical pesticides are currently the main method for controlling diseases and insect pests; however, their excessive use has caused serious environmental, human, and animal safety problems. While breeding disease-resistant varieties can be an alternative solution, the process is time-consuming and labor-intensive. Consequently, there are challenges in meeting the urgent production demands. Nonetheless, in this context, the research and development of biopesticides using RNA-silencing technology have proven highly beneficial. These novel pesticides, characterized by strong specificity and environmental friendliness, including insecticides, fungicides, and herbicides, provide an innovative approach to sustainable plant protection and development [95,96]. Considering the crucial role of miRNAs in animals and plants, miRNA biopesticides are essential for future pest control. It is important to ensure that the target miRNA effectively acts on the intended organism to make use of this technology in controlling diseases and insect pests. Currently, there are two main methods of miRNA delivery. The first is “host-induced gene silencing”, which involves using transgenic or gene-editing techniques to make the plant endogenously express the target miRNA fragment. When the pathogen infects, or the insect feeds on the transgenic plant, the miRNA enters the harmful organism to perform its function. The second method, known as “spray-induced gene silencing”, involves directly applying fungicides or insecticides to crops to stabilize the synthesized functional miRNA in vitro [97,98]. Recently, numerous biological pesticides based on RNAi have been approved for field use and have demonstrated promising application prospects.

In modern plant protection strategies, miRNA spray technologies offer a promising approach to enhancing plant resistance to pests. miRNAs are small non-coding RNA molecules that regulate gene expression and play a vital role in plant defense mechanisms. For example, miRNA spray technologies have effectively deterred pest feeding in genetically modified crops, resulting in improved yield and quality [99].

Recent advancements in nanotechnology have introduced new delivery systems for miRNAs. Specifically, carbon quantum dots and carbon nanotubes have shown great potential as delivery vehicles in plant research. Carbon quantum dots are especially advantageous because they are highly biocompatible and have low toxicity. This makes them ideal for delivering small interfering RNAs (siRNAs) and miRNAs. Schwartz et al. demonstrated the effectiveness of carbon quantum dots in delivering siRNAs and silencing genes in plants, which supports the further development of miRNA spray technologies. Additionally, Demirer et al. showed that carbon nanotubes can deliver functional genetic material to plants without integrating transgenes, which highlights their broad application potential in plant genetic engineering [100,101].

These nanomaterials not only improve the stability and bioavailability of miRNAs but also overcome the limitations of traditional delivery methods. They offer a more efficient and environmentally friendly solution for pest control in agriculture [102,103].

However, developing commercial disease-resistant varieties for all plants through transgenic technology is challenging, as is the simultaneous control of multiple diseases and insect pests. Other challenges in applying this technology include the development of stable and efficient exogenous RNA-based biological pesticides in the field and the high production cost involved. Additionally, to avoid unintended effects and potential safety risks, the sequence design of miRNAs and target genes must undergo rigorous scientific screening.

**Figure 2:**
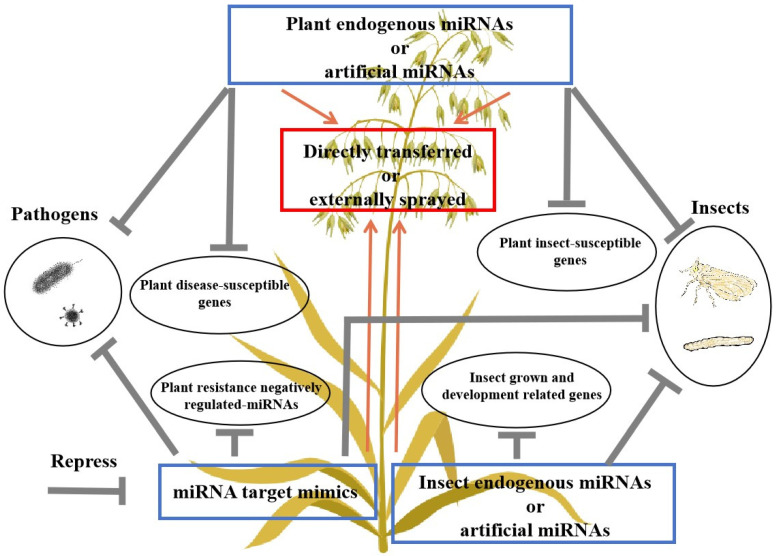
Functions of exogenous and endogenous miRNAs and amiRNAs in disease and insect resistance in plants. MiRNAs, miRNA target mimetics, and amiRNAs related to plant disease and pest resistance can be synthesized externally or directly transferred into the crop genome to enhance crop disease and pest resistance.

The main technical route for cultivating disease- and insect-resistant plants using miRNAs, miRNA target mimics, or amiRNAs is shown in Figure 2.

### 5.2. Challenges in the Application of miRNA Technology in Crop Breeding

With the advancement and refinement of high-throughput sequencing technologies, numerous miRNAs associated with stress response and development have been identified in plants, including some involved in regulating programmed cell death (PCD). However, there are still many issues that require further investigation. Currently, research on the direct regulation of PCD by miRNAs is mainly focused on animals and humans, with relatively few studies conducted in plants. Therefore, more plant-focused studies are needed. Additionally, many miRNA functions predicted using bioinformatics require further validation, particularly in plants. It is crucial to analyze the function of miRNA structures first, as a conserved miRNA structure often indicates a conserved function. The regulatory functions of the same type of miRNA may vary in different plants, under different stress conditions, and at different reproductive stages. Exploring the mechanisms by which miRNAs regulate PCD can be achieved by studying the relationship between miRNAs and PCD-related genes that respond to environmental stress, regulate development, and induce PCD. Although miRNAs are known to be closely associated with hormone, calcium, and reactive oxygen species signals in plant PCD, the upstream and downstream relationships among them are still unclear. Determining whether miRNAs play pivotal roles in these processes is crucial for understanding the mechanisms of action in plant PCD. However, despite the promising potential of miRNA technology in enhancing disease resistance in plants, there are significant challenges. One major challenge is the durability and stability of the transgene. The expression of miRNAs can be unstable under different environmental conditions, which may affect their long-term effectiveness in crops. Furthermore, there are concerns about the potential negative impacts of transgenic miRNAs on consumer health. These issues arise from an insufficient understanding of miRNA mechanisms, particularly in plants. Therefore, further research on miRNA function in plants is critical for developing safer, more stable, and effective miRNA-based technologies. Further studies on the application of plant miRNAs are needed to improve crop stress resistance and yield. Approaches such as miRNA overexpression or silencing, overexpression of miRNA target sites, mutating target genes, and using synthetic miRNAs can help clarify the molecular mechanisms of plant adaptation to stress and the regulation of growth and development, providing new strategies for improving disease resistance, stability, and safety in crops.

Although the role of miRNAs in insect resistance has yet to be extensively studied, their applications in pest control have been demonstrated. The discovery of RNAi and its associated regulatory mechanisms has inspired researchers to pursue crop pest management and control. However, developing new green pesticides and insecticidal technology remains an urgent problem in ensuring agricultural safety. Studies have shown that targeting key genes involved in the growth and development of pests using RNAi can cause abnormal growth and reproduction, ultimately leading to their demise. Currently, RNAi technology is recognized as the most promising method for developing environmentally friendly pesticides. To apply this technology to agricultural production, it is crucial to identify the key target genes involved in pest growth and reproduction and establish a safe and effective application system.

While RNAi offers greater specificity and biological safety compared to other pest control strategies, RNAi-based products and their applications still encounter several challenges. For instance, the high specificity of these products limits their effectiveness against a wide range of insects, thus narrowing their scope in pest control. Furthermore, pest control based on RNAi technology is slower than traditional chemical control methods. The response levels of different pest populations may vary, and there is also the potential for miRNAs to have off-target effects.

### 5.3. Conclusions

In conclusion, the role of miRNA in biotic stress has been studied, although not as extensively as their involvement in abiotic stress. Studies have shown that when plants are attacked by external pathogens or insects, many miRNAs influence the plant’s response by regulating the expression of relevant genes in resistance pathways. Further research on the relationship between miRNAs and plant stress responses will aid in developing new plant varieties with stronger stress resistance. In recent years, there has been an increasing number of studies on miRNAs in response to various plant stresses. With ongoing scientific and technological advancements, the mechanisms by which miRNAs and their target genes regulate plant stress responses have become clearer. However, miRNAs belong to a large family, have numerous target genes, and involve complex regulatory mechanisms. Functional verification of miRNAs in response to stress and their participation in the molecular regulatory network of plant stress responses is crucial for ensuring plant resistance to various stressors. A comprehensive understanding of the mechanisms and molecular regulatory network pathways of miRNAs and their target genes involved in plant stress responses is needed. Furthermore, studying the role of miRNA in the molecular mechanisms of resistance to plant diseases and insects will aid in cultivating disease- and insect-resistant plants.

## Figures and Tables

**Table 1:** Known functions of miRNAs in the development of resistance to insects in plants.

miRNA	Target(s)	Plant Species	Insect Species	Function	Reference
AtmiR156	*SPL9*	*A. thaliana*	*Helicoverpa armigera Plutella xylostella*	Decrease weight and prolong larval stage	[8]
Ghr-miR166b	*ATP synthase gene*	*Gossypium hirsutum*	*Bemisia tabaci*	Death	[9]
miR-24	*Chitinase*	*N. tabacum*	*H. armigera*	Death	[10]
OsmiR156	*SPL14*	*O. sativa*	*Nilaparvata lugens*	Decrease fertility	[11]
OsmiR159	*OsGAMYBL2*	*O. sativa*	*N. lugens*	Decrease honeydew excretion	[12]
OsmiR396	*OsGRF8*	*O. sativa*	*N. lugens*	Decrease honeydew excretion	[13]
OsmiR162a	*OsDCL1*	*O. sativa*	*N. lugens*	Decrease fertility	[14]
PtmiR319a	*TCP*	*Populus tomentosa*	*Spodoptera frugiperda*	Decrease feeding	[15]
SlmiR319	*TCP4*	*Solanum lycopersicum*	*Meloidogyne incognita*	Decrease fertility	[16]

**Table 2:** Known functions of miRNAs in the development of plant resistance to diseases.

miRNA	Target(s)	Plant Species	Pathogen Species	Function	Reference
AtmiR393	*TIR1*	*A. thaliana*	*Pseudomonas syringae*	Increase resistance	[38]
GhmiR2118	TIR-NBS-LRR genes	*G. hirsutum*	*Verticillium dahliae*	Promote target protein accumulation, enhance root defense	[39]
GhmiR166, GhmiR159	*Clp-1*, *HiC-15*	*G. hirsutum*	*V. dahliae*	Identification and degradation	[40]
GhmiR164	*GhNAC100*	*G. hirsutum*	*V. dahliae*	Increase resistance	[41]
NtmiR482b	Solyc02g036270.2	*N. tabacum*	*Phytophthora parasitica*	Increase NBS-LRR expression and resistance	[42]
OsmiR168	*Ago1*	*O. sativa*	*Magnaporthe oryzae*	Enhance resistance	[43]
OsmiR395	ATP sulfurylase gene *OsAPS1*	*O. sativa*	*Xanthomonas oryzae* pv. *oryzae* (Xoo); *X. oryzae* pv. *oryzicola* (Xoc)	Enhance broad-spectrum resistance.	[44]
OsmiR398b	Superoxide dismutase	*O. sativa*	*M. oryzae*	Upregulate of H_2_O_2_ level, increase resistance	[34]
OsmiR528	L-Ascorbate oxidase	*O. sativa*	*Rice stripe tenuivirus*	Reduce cell death and damage	[37]
StmiR482, SlmiR482	Ry, NL25, N, RB, R2, and R3a	*Solanum tuberosum*, *S. lycopersicum*	*Phytophthora infestans*	Genes cluster to confer resistance	[45]
PN-2013	Monodehydroascorbate reductase gene	*Triticum aestivum*	*Puccinia striiformis*	Upregulate PR gene expression, increase resistance	[36]
ZmmiR168	*Ago1*	*Z. mays*	*Rhizoctonia solani*	Promote resistance gene accumulations, increase resistance	[46]

**Table 3:** Known functions of amiRNAs in developing resistance to insects and diseases in plants.

Target(s)	Plant Species	Insect/Pathogen Species	Function	Reference
HC-Pro, p69 gene	*A. thaliana*	Turnip mosaic virus, Turnip yellow mosaic virus	Mediate viral RNA cleavage, Increase resistance	[73]
ATP synthase gene	*G. hirsutum*	*Bemisia Tabaci*	Death	[9]
2b gene	*Lycopersicum esculentummill*	Cucumber mosaic virus	Target viral RNA, Increase resistance	[74]
HaAce1 gene	*N. tabacum*	*H. armigera*	Induce larval death and deformity in adult	[75]
2b gene	*N. tabacum*	Cucumber mosaic virus	Target viral RNA, Increase resistance	[76]
Nuclear inclusion b protein, coat protein	*N. tabacum*	Potato virus Y	Target viral RNA, Increase resistance	[77]
MpAChE2	*N. tabacum*	*M. persicae*	Decrease fecundity	[28]
Ecdysone receptor, Spook gene	*O. sativa*	*Chilo suppressalis*	Death	[24]
Disembodied protein	*O. sativa*	*C. suppressalis*	Death	[78]
DN70206_c1_g10	*O. sativa*	*C. suppressalis*	Decrease weight, prolong larval stage	[79]
DN90065_c0_g12	*O. sativa*	*C. suppressalis*	Decrease weight, decrease eclosion rate, prolong larval stage	[80]
*Xa13*	*O. sativa*	*X. oryzae*	Silence genes, increase resistance	[81]
HC-Pro, p25 gene	*S. tuberosum*	Potato virus Y	Reduce pathogenicity	[70]
HC-Pro, p25 gene	*S. tuberosum*	Potato virus X	Reduce pathogenicity	[70]
